# Association Between Periodontitis and Metabolic Dysfunction–Associated Steatotic Liver Disease: A Cross‐Sectional Study

**DOI:** 10.1002/cre2.70355

**Published:** 2026-04-08

**Authors:** Mizuki Saito, Yoshihiro Shimazaki, Saori Yoshii, Tetsuhito Kojima

**Affiliations:** ^1^ Department of Preventive Dentistry and Dental Public Health, School of Dentistry Aichi Gakuin University Nagoya Japan; ^2^ Aichi Health Promotion Foundation Nagoya Japan

**Keywords:** metabolic dysfunction–associated steatotic liver disease, periodontitis, sex difference

## Abstract

**Objective:**

This study investigated the association between periodontitis and metabolic dysfunction–associated steatotic liver disease (MASLD), with particular attention paid to sex differences.

**Material and Methods:**

Participants aged 40–74 years who underwent health examinations were included. Logistic regression analyses were conducted with MASLD as the dependent variable and periodontitis and other variables as independent variables. Odds ratios (ORs) and 95% confidence intervals (CIs) were calculated separately for men and women. The women were divided into three age groups (40–49, 50–59, and 60–74 years) to account for the influence of sex hormones. Interaction analyses were performed to examine sex differences in the association between periodontitis and MASLD.

**Results:**

The OR for MASLD was significantly higher for women with moderate or severe periodontitis than for those without periodontitis [1.40 (95% CI: 1.04–1.89) and 1.64 (95% CI: 1.04–2.59)]. Periodontitis was associated significantly with MASLD among women aged 50–59 years, but not among women in other age groups or men. A significant multiplicative, but not additive, interaction between sex and periodontitis was found to affect the risk of MASLD.

**Conclusions:**

Periodontitis was associated with MASLD in women. The association of periodontitis with MASLD may differ between men and women.

## 1 Introduction

Steatotic liver disease is the most common chronic liver condition, affecting approximately 32% of adults worldwide (Maurice and Manousou 2018). It can progress to hepatocellular carcinoma and subsequently to cirrhosis (Maurice and Manousou 2018). Hepatocellular carcinoma carries a poor prognosis among cancers due to its high relapse rate and low survival (World Health Organization International Agency for Research on Cancer 2022). Moreover, because steatotic liver disease increases the risk of stroke, ischemic heart disease, and other fatal conditions (Younossi 2019), prevention of its onset and progression is of critical importance.

Non‐alcoholic fatty liver disease (NAFLD) was previously used to describe steatotic liver disease. However, in 2023, the terminology was revised to metabolic dysfunction–associated steatotic liver disease (MASLD) because the term NAFLD did not accurately capture the underlying pathology, and the terms “alcoholic” and “fatty” are considered stigmatizing (Rinella et al. 2023). In diagnosing MASLD, not only alcohol consumption but also metabolic abnormalities such as obesity, diabetes, and hypertension are taken into account (Rinella et al. 2023).

Lifestyle habits are closely linked to the incidence and progression of steatotic liver disease, and several studies have reported periodontal disease to be a risk factor for its development (Kuraji et al. 2021; Tan et al. 2024). An 11‐year follow‐up study using medical insurance data demonstrated that periodontal disease increased the risk of NAFLD onset (Shin et al. 2022). Moreover, a study of patients with NAFLD indicated that periodontal disease may influence the progression of liver fibrosis (Kuroe et al. 2021). Collectively, these findings highlight a growing body of evidence on the relationship between periodontal disease and NAFLD. However, few studies have examined the association between periodontal disease and MASLD (Shine et al. 2024; Zhang et al. 2025), as MASLD was only recently redefined. NAFLD is considered a hepatic phenotype of metabolic syndrome, and individuals with NAFLD frequently present with concomitant metabolic abnormalities such as hyperglycemia, hypertension, and dyslipidemia. Consequently, 98% concordance between NAFLD and MASLD has been reported (Suzuki et al. 2024), although the diagnosis of MASLD requires the presence of metabolic abnormalities. Nevertheless, this study was conducted in NAFLD patients receiving treatment at medical institutions, and did not account for individuals with NAFLD who did not seek medical care. Therefore, uncertainty remains as to whether findings from NAFLD studies can be directly extrapolated to MASLD (Younossi et al. 2024).

This study investigated the association between periodontitis and MASLD in individuals undergoing annual health checkups. In Japan, the prevalence of NAFLD is higher in men than in women, and periodontal disease has also been reported to be more common in men (Eguchi et al. 2012; Ministry of Health, Labour and Welfare 2022). Sex is an important factor in health research, as differences exist not only in disease prevalence but also in health awareness and lifestyle behaviors. Research involving both humans and animals has emphasized the importance of considering sex as a key factor (Heidari et al. 2016). Accordingly, this study also evaluated sex differences in the association between periodontitis and MASLD.

## 2 Methods

### 2.1 Participants

This study included individuals aged 40–74 years who underwent comprehensive health examinations, including oral health assessments, at the Aichi Health Promotion Foundation between April 1, 2014 and March 31, 2015. Participants with a history of liver disease, hepatitis B surface antigen positivity, alcohol consumption ≥ 20 g/day, or no teeth subject to periodontal evaluation were excluded. Written informed consent to use health examination data for research was obtained from all participants. The Institutional Review Board of Aichi Gakuin University School of Dentistry approved the study design, data collection methods, and informed consent procedure (no. 618). This study was reported and performed according to the Strengthening the Reporting of Observational Studies in Epidemiology guidelines.

### 2.2 Health Examinations

As part of the health checkups, measurements of height, weight, waist circumference, and blood pressure were recorded, and blood biochemistry tests along with abdominal ultrasound were performed. The blood tests provided data on blood glucose (mg/dL), HbA1c (%), triglycerides (mg/dL), high‐density lipoprotein (HDL) cholesterol (mg/dL), low‐density lipoprotein (LDL) cholesterol (mg/dL), and alanine aminotransferase (ALT) (U/L). Abdominal ultrasound findings were used to diagnose steatotic liver disease. The ultrasound was performed by clinical laboratory technicians, and diagnoses were confirmed by gastroenterologists.

### 2.3 MASLD

Participants with steatotic liver disease identified on abdominal ultrasound who met at least one of the five cardiometabolic criteria were diagnosed with MASLD. The cardiometabolic criteria were defined as follows: obesity, body mass index ≥ 23 kg/m² or waist circumference >94 cm in men and >80 cm in women; diabetes, blood glucose ≥100 mg/dL, HbA1c ≥ 5.7%, or a diagnosis/treatment of type 2 diabetes; hypertension, blood pressure ≥130/85 mmHg, or use of antihypertensive medication; hyperlipidemia, triglycerides ≥150 mg/dL or lipid‐lowering therapy; and low HDL cholesterol, HDL ≤ 40 mg/dL in men and ≤50 mg/dL in women, or lipid‐lowering therapy (Rinella et al. 2023).

### 2.4 Oral Health Examinations

As part of the oral health examination, tooth condition and periodontal status were assessed. Each tooth was recorded as sound, decayed, filled, or missing, and the total number of sound, decayed, and filled teeth (excluding third molars) was calculated as the number of teeth present. Periodontal status was evaluated using the community periodontal index (CPI) (World Health Organization 1997). The oral cavity of each participant was divided into sextants, and 10 representative teeth (11, 16, 17, 26, 27, 31, 36, 37, 46, and 47) were examined. Measurements were obtained with a CPI probe (YDM Co., Tokyo, Japan) at six sites per tooth (mesio‐buccal, mid‐buccal, disto‐buccal, disto‐lingual, mid‐lingual, and mesio‐lingual). CPI codes were defined as follows: code 0, healthy; code 1, bleeding on probing; code 2, dental calculus detected; code 3, periodontal pockets of 4–5 mm; and code 4, periodontal pockets ≥6 mm. Sextants without representative teeth were coded as X. The highest CPI code among sextants, excluding code X, was recorded as the individual CPI code. Examinations were performed by 12 dentists, each calibrated with an inter‐examiner kappa index of ≥ 0.5, under reflected light using a dental mirror.

### 2.5 Questionnaire

A self‐administered questionnaire was used to collect information on participants’ smoking habits, alcohol consumption, exercise, eating speed, and medical history. Smoking status was categorized as never, past, or current. Alcohol consumption was categorized as daily intake of 0, <20, ≥20–39, ≥40–59, or ≥60 g. Exercise habits were evaluated with the question “Have you engaged in exercise that caused light sweating for at least 30 min per session, twice weekly, for more than 1 year?” with response options of yes or no. Eating speed was classified as fast, normal, or slow.

### 2.6 Statistical Analyses

Periodontitis was classified into three categories based on the individual CPI code: codes 0, 1, and 2 were defined as no periodontitis, code 3 was defined as moderate periodontitis, and code 4 was defined as severe periodontitis.

To assess the association between each variable and MASLD, *t*‐tests were used to compare the mean values and chi‐squared tests were used to compare proportions. Logistic regression analyses were conducted to evaluate the association between periodontitis and MASLD, with odds ratios (ORs) and 95% confidence intervals (CIs) calculated separately for men and women. MASLD was the dependent variable and factors suggested to be associated with MASLD (age, smoking status, alcohol consumption, exercise, eating speed, ALT level, LDL cholesterol level, and periodontitis) were included as independent variables (Kolay et al. 2021; Tamber et al. 2023; Yan et al. 2024; Younossi et al. 2025). Although body mass index, blood glucose levels, blood pressure, and blood lipids are associated with periodontitis and liver disease (Genco and Borgnakke 2013; Huang et al. 2020), these factors are included in the diagnostic criteria for MASLD and were therefore not used in the analyses. As female sex hormones significantly influence women's health, with declines in these hormones in the age range of the study participants increasing the risk of lifestyle‐related diseases (Eng et al. 2023), female participants were divided into three age groups (40–49, 50–59, and 60–74 years).

As the exclusion criterion for alcohol consumption used in this study was stricter than the standard cut‐off value of 30 g/day for men, the potential impact of selection bias was assessed by performing a logistic regression analysis of the association between periodontitis and MASLD in non‐drinkers.

We investigated the interaction between periodontitis and sex on the risk of MASLD. Periodontitis was classified as “no” or “moderate to severe.” Multiplicative scale, relative excess risk due to interaction (RERI), attributable proportion (AP), and synergy index (SI) values were calculated, with women without periodontitis serving as the reference group. The interaction analyses were conducted with R (version 4.2.1). All other statistical analyses were conducted with SPSS (version 28.0; IBM Corporation, Armonk, NY, USA). The significance level was set to *p* < 0.05.

## 3 Results

A total of 13,788 individuals underwent physical and oral health examinations during the study period, of whom 11,850 were aged 40–74 years. After the exclusion of participants with hepatitis B surface antigen positivity (*n* = 353), a history of liver disease (*n* = 316), alcohol consumption >20 g/day (*n* = 4956), no teeth subject to periodontal evaluation (*n* = 31), and missing data (*n* = 10), 6184 individuals were included in the analysis. Among them, 1431 (23.1%) were diagnosed with MASLD.

Table 1 presents the associations between each variable and MASLD. The ALT level, LDL cholesterol level, sex, smoking, alcohol consumption, exercise, eating speed, and periodontitis were associated significantly with MASLD.

**Table 1:** Characteristics of participants according to MASLD.

	MASLD		
Variable	Absence (*n* = 4753)	Presence (*n* = 1431)	*p*
	Mean (SD)		
Age (years)	52.3 (8.9)	52.1 (8.5)	0.372
ALT (U/L)	20.5 (12.9)	35.4 (22.9)	< 0.001
LDL cholesterol (mg/dL)	127.1 (29.3)	138.9 (31.0)	< 0.001
	*n* (%)		
Sex			
Men	2857 (60.1)	1166 (81.5)	< 0.001
Women	1.896 (39.9)	265 (18.5)	
Smoking			
Never	3,088 (65.5)	784 (54.8)	< 0.001
Past	746 (15.6)	301 (21.0)	
Current	922 (19.4)	346 (24.2)	
Alcohol consumption			
No	2656 (55.9)	859 (60.0)	0.003
Yes	2097 (44.1)	572 (40.0)	
Exercise			
No	3696 (77.8)	1187 (82.9)	< 0.001
Yes	1057 (22.2)	244 (17.1)	
Eating speed			
Normal	2802 (59.0)	710 (49.6)	< 0.001
Slow	374 (7.9)	53 (3.7)	
Fast	1577 (33.2)	668 (46.7)	
Periodontitis			
No	2210 (46.5)	516 (36.1)	< 0.001
Moderate	1941 (40.8)	677 (47.3)	
Severe	602 (12.7)	238 (16.6)	

Abbreviations: ALT, alanine aminotransferase; LDL cholesterol, low‐density lipoprotein cholesterol; MASLD, metabolic dysfunction–associated steatotic liver disease; SD, standard deviation.

Tables 2 and 3 show the results of logistic regression analyses conducted separately for men and women regarding the association between periodontitis and MASLD. Among men, ALT level, LDL cholesterol level, alcohol consumption, exercise, and eating speed were associated significantly with MASLD (Table 2). The association between periodontitis and MASLD was significant in the crude model, but not after adjustment. Among women, age, ALT level, LDL cholesterol level, eating speed, and periodontitis were associated significantly with MASLD (Table 3). Women with moderate and severe periodontitis had significantly higher ORs for MASLD compared with those without periodontitis [1.40 (95% CI: 1.04–1.89) and 1.64 (95% CI: 1.04–2.59)].

**Table 2:** Association between MASLD and independent variables by logistic regression analysis in men.

	MASLD		Dependent variable: MASLD (absence = 0, presence = 1)			
Independent variable	Absence (*n* = 2857)	Presence (*n* = 1166)	Crude OR (95% CI)	*p*	Adjusted OR (95% CI)	*p*
	Mean (SD)					
Age (years)	52.1 (8.9)	51.3 (8.3)	0.99 (0.98–1.00)	0.009	1.01 (1.00‐1.02)	0.169
ALT (U/L)	22.9 (13.7)	36.9 (23.4)	1.05 (1.05–1.06)	<0.001	1.05 (1.04‐1.05)	<0.001
LDL cholesterol (mg/dL)	129.6 (29.1)	139.1 (31.1)	1.01 (1.01–1.01)	<0.001	1.01 (1.01‐1.01)	<0.001
	*n* (%)					
Smoking						
Never	1389 (48.6)	553 (47.4)	1		1	
Past	646 (22.6)	286 (24.5)	1.11 (0.94–1.32)	0.222	1.12 (0.93–1.35)	0.252
Current	822 (28.8)	327 (28.0)	1.00 (0.85–1.18)	0.992	0.99 (0.83–1.19)	0.914
Alcohol consumption						
No	1,418 (49.6)	672 (57.6)	1		1	
Yes	1439 (50.4)	494 (42.4)	0.73 (0.63–0.83)	<0.001	0.78 (0.67–0.91)	0.001
Exercise						
No	2,204 (77.1)	974 (83.5)	1		1	
Yes	653 (22.9)	192 (16.5)	0.67 (0.56‐0.80)	<0.001	0.75 (0.62–0.91)	0.003
Eating speed						
Normal	1578 (55.2)	549 (47.1)	1		1	
Slow	210 (7.4)	43 (3.7)	0.59 (0.42–0.83)	0.002	0.68 (0.47–0.97)	0.033
Fast	1,069 (37.4)	574 (49.2)	1.54 (1.34‐1.78)	<0.001	1.37 (1.17–1.59)	<0.001
Periodontitis						
No	1092 (38.2)	394 (33.8)	1		1	
Moderate	1312 (45.9)	568 (48.7)	1.20 (1.03–1.40)	0.018	1.11 (0.94–1.31)	0.238
Severe	453 (15.9)	204 (17.5)	1.25 (1.02–1.53)	0.031	1.21 (0.97–1.52)	0.098

Abbreviations: ALT, alanine aminotransferase; CI, confidence interval; LDL cholesterol, low‐density lipoprotein cholesterol; MASLD, metabolic dysfunction–associated steatotic liver disease; OR, odds ratio; SD, standard deviation.

**Table 3:** Association between MASLD and independent variables by logistic regression analysis in women.

	MASLD		Dependent variable: MASLD (absence = 0, presence = 1)			
Independent variable	Absence (*n* = 1896)	Presence (*n* = 265)	Crude OR (95% CI)	*p*	Adjusted OR (95% CI)	*p*
	Mean (SD)					
Age (years)	52.7 (8.8)	55.5 (8.7)	1.04 (1.02–1.05)	<0.001	1.02 (1.00–1.04)	0.048
ALT (U/L)	17.0 (10.7)	28.4 (19.2)	1.06 (1.05–1.07)	<0.001	1.05 (1.04–1.06)	<0.001
LDL cholesterol (mg/dL)	123.4 (29.2)	138.1 (30.6)	1.02 (1.01–1.02)	<0.001	1.01 (1.01–1.02)	<0.001
	*n* (%)					
Smoking						
Never	1.699 (89.6)	231 (87.2)	1		1	
Past	97 (5.1)	15 (5.7)	1.14 (0.65–1.99)	0.653	1.22 (0.66–2.25)	0.530
Current	100 (5.3)	19 (7.2)	1.40 (0.84–2.33)	0.198	1.45 (0.83–2.53)	0.194
Alcohol consumption						
No	1,238 (65.3)	187 (70.6)	1		1	
Yes	658 (34.7)	78 (29.4)	0.79 (0.59–1.04)	0.090	0.87 (0.64–1.18)	0.377
Exercise						
No	1,492 (78.7)	213 (80.4)	1		1	
Yes	404 (21.3)	52 (19.6)	0.90 (0.65–1.25)	0.529	0.84 (0.59–1.20)	0.333
Eating speed						
Normal	1,224 (64.6)	161 (60.8)	1		1	
Slow	164 (8.6)	10 (3.8)	0.46 (0.24–0.90)	0.022	0.46 (0.23–0.95)	0.035
Fast	508 (26.8)	94 (35.5)	1.41 (1.07–1.85)	0.015	1.36 (1.01–1.82)	0.040
Periodontitis						
No	1,118 (59.0)	122 (46.0)	1		1	
Moderate	629 (33.2)	109 (41.1)	1.59 (1.21–2.09)	0.001	1.40 (1.04–1.89)	0.028
Severe	149 (7.9)	34 (12.8)	2.09 (1.38–3.17)	<0.001	1.64 (1.04–2.59)	0.035

Abbreviations: ALT, alanine aminotransferase; CI, confidence interval; LDL cholesterol, low‐density lipoprotein cholesterol; MASLD, metabolic dysfunction–associated steatotic liver disease; OR, odds ratio; SD, standard deviation.

Table 4 shows the results of the logistic regression analyses for women in different age groups to account for the influence of sex hormones. In the 50–59‐year‐old group, a significant association was observed between periodontitis and MASLD. The OR for MASLD was significantly higher for women without periodontitis than for those with moderate to severe periodontitis [1.71 (95% CI: 1.04–2.79) and 2.33 (95% CI: 1.15–4.73)]. In the 40–49‐ and 60–74‐year‐old groups, no significant association was observed between periodontitis and MASLD.

**Table 4:** Association between MASLD and periodontitis by logistic regression analysis in women according to age groups.

		Dependent variable: MASLD (absence = 0, presence = 1)			
Variable	*n*	Crude OR (95% CI)	*p*	Adjusted OR* (95% CI)	*p*
40–49 years old					
Periodontitis					
No	609	1		1	
Moderate	232	1.41 (0.85–2.35)	0.182	1.50 (0.85–2.62)	0.159
Severe	26	0.98 (0.22–4.25)	0.974	1.10 (0.24–5.08)	0.906
50–59 years old					
Periodontitis					
No	400	1		1	
Moderate	275	1.85 (1.17–2.93)	0.008	1.71 (1.04–2.79)	0.033
Severe	49	2.60 (1.36–4.96)	0.004	2.33 (1.15–4.73)	0.020
60–74 years old					
Periodontitis					
No	231	1		1	
Moderate	231	1.14 (0.69–1.87)	0.612	1.03 (0.60–1.77)	0.920
Severe	84	1.30 (0.68–2.49)	0.432	1.23 (0.60–2.53)	0.564

Abbreviations: CI, confidence interval; MASLD, metabolic dysfunction–associated steatotic liver disease; OR, odds ratio.

* Adjusted for age, alanine aminotransferase, low‐density lipoprotein cholesterol, smoking, alcohol consumption, exercise, and eating speed.

Table 5 presents the results of a logistic regression analysis examining the association between periodontitis and MASLD in non‐drinkers that was conducted to eliminate the influence of alcohol consumption. Among non‐drinkers, a significant association between periodontitis and MASLD was observed for women but not for men. This trend was similar to the results obtained for mild drinkers. The ORs for MASLD were 1.76 (95% CI: 1.22–2.55) for women with moderate periodontitis and 1.93 (95% CI: 1.11–3.36) for those with severe periodontitis relative to women without periodontitis.

**Table 5:** Association between MASLD and independent variables by logistic regression analysis in non‐drinkers.

	Dependent variable: MASLD (absence = 0, presence = 1)			
	Men		Women	
Independent variable	Adjusted OR (95% CI)	*p*	Adjusted OR (95% CI)	*p*
Age (years)	1.01 (1.00–1.02)	0.168	1.01 (0.99–1.03)	0.228
ALT (U/L)	1.05 (1.04–1.05)	<0.001	1.07 (1.05–1.08)	<0.001
LDL cholesterol (mg/dL)	1.01 (1.00‐1.01)	<0.001	1.01 (1.01–1.02)	<0.001
Smoking				
Never	1		1	
Past	1.25 (0.96–1.62)	0.096	1.53 (0.71–3.31)	0.276
Current	1.06 (0.84–1.34)	0.634	1.40 (0.71–2.75)	0.335
Exercise				
No	1		1	
Yes	0.66 (0.51–0.86)	0.002	0.83 (0.52–1.32)	0.429
Eating speed				
Normal	1		1	
Slow	0.79 (0.49–1.26)	0.320	0.52 (0.21–1.25)	0.144
Fast	1.28 (1.05–1.58)	0.017	1.43 (1.00–2.05)	0.050
Periodontitis				
No	1		1	
Moderate	1.08 (0.86–1.36)	0.500	1.76 (1.22–2.55)	0.003
Severe	1.13 (0.84–1.53)	0.427	1.93 (1.11–3.36)	0.021

Abbreviations: ALT, alanine aminotransferase; CI, confidence interval; LDL cholesterol, low‐density lipoprotein cholesterol; MASLD, metabolic dysfunction–associated steatotic liver disease; OR, odds ratio.

Table 6 presents the results of the analysis of the interaction of sex and periodontitis on MASLD. The multiplicative scale (an indicator of multiplicative interaction) was significant, but the RERI, AP, and SI (indicators of additive interaction) were not. The multiplicative scale value was 0.72 (95% CI: 0.53–0.99), the RERI value was –0.28 (95% CI: –0.39–0.19), the AP value was –0.11 (95% CI: –0.34–0.08), and the SI value was 0.85 (95% CI: 0.63–1.13).

**Table 6:** Interactive effect of sex and periodontitis on MASLD by logistic regression analysis.

	Dependent variable: MASLD (absence = 0, presence = 1)		
	No periodontitis OR (95% CI)	Moderate or severe periodontitis OR (95% CI)	Effect of periodontitis within the strata of sex OR (95% CI)
Women	1 (reference)	1.55 (1.18–2.04)	1.55 (1.18–2.04)
Men	2.26 (1.78–2.87)	2.53 (2.01–3.19)	1.12 (0.96–1.31)
Effect of sex within the strata of periodontitis	2.26 (1.78–2.87)	1.63 (1.31–2.04)	
Multiplicative scale	0.72 (0.53–0.99)		
RERI	−0.28 (−0.93–0.19)		
AP	−0.11 (−0.34–0.08)		
SI	0.85 (0.63–1.13)		

*Note:* Adjusted for age, alanine aminotransferase, low‐density lipoprotein cholesterol, smoking, alcohol consumption, exercise, and eating speed.

Abbreviations: AP, attributable proportion; CI, confidence interval; MASLD, metabolic dysfunction–associated steatotic liver disease; OR, odds ratio; RERI, relative excess risk due to interaction; SI, synergy index.

## 4 Discussion

In this study, ORs for MASLD were significantly higher for women with periodontitis than for those without periodontitis. This sex‐specific association and the detection of significant multiplicative interaction imply that the association between periodontitis and MASLD differs between women and men.

As few studies have investigated the association between periodontitis and MASLD, the findings of this study are valuable. In particular, the sex differences observed in the association warrant further investigation. However, several limitations should be considered. First, because this was a cross‐sectional study, causal relationships and underlying mechanisms could not be established. Second, periodontitis was assessed using a partial examination method, which may have led to underestimation or misclassification of disease severity. Third, steatotic liver disease was diagnosed using abdominal ultrasonography rather than liver biopsy, the gold standard. Fourth, socioeconomic factors such as education and economic status, which influence lifestyle and health outcomes (Williams et al. 2018), were not available and thus could not be accounted for. Fifth, MASLD exclusion criteria based on alcohol consumption are defined as 140 g/week for women and 210 g/week for men; however, the questionnaire in this study only allowed for an estimate of 20 g/day, applied uniformly to both sexes. As a result, more men were excluded based on alcohol intake, potentially biasing the male sample toward healthier individuals. To address this issue, the association between periodontitis and MASLD in non‐drinkers was examined. Similar to the results obtained for mild drinkers, this association was significant only in women, implying that the impact of using a non‐standard alcohol consumption criterion was minimal. Sixth, information on smoking, alcohol consumption, exercise, and eating speed was obtained through a self‐reported questionnaire and may not have accurately reflected participants’ actual behaviors. Finally, because participants were limited to those undergoing health examinations at a single facility in Japan, the findings may not be generalizable to other populations.

Several studies have examined the association between periodontitis and NAFLD (Kuroe et al. 2021; Sato et al. 2022; Shin et al. 2022; Weintraub et al. 2019; Yoneda et al. 2012). A cross‐sectional study reported that individuals with tooth loss, periodontitis, and dental caries had higher ORs for NAFLD (Weintraub et al. 2019). A longitudinal study demonstrated that periodontitis increased the risk of incident NAFLD (Shin et al. 2022). In a case–control study, NAFLD patients showed a higher rate of Porphyromonas gingivalis in saliva, with the highest rate observed in patients with non‐alcoholic steatohepatitis (Yoneda et al. 2012). Several reports have described the association between periodontal disease and MASLD (Shine et al. 2024; Zhang et al. 2025), but no study has investigated sex differences in this association. Our study extends this evidence to MASLD, showing that severe periodontitis was significantly associated with higher ORs of MASLD. Furthermore, the findings imply that the association of periodontitis with MASLD differed between men and women.

The most likely explanation for the significant association between periodontitis and MASLD in this study is that the two conditions share common risk factors. Stress and lifestyle habits such as smoking and poor diet are established risk factors for both periodontal disease and steatotic liver disease (Genco and Borgnakke 2013; Younossi et al. 2023). Oral health behaviors, including regular toothbrushing and dental visits, are essential for maintaining good periodontal health (Pham et al. 2011). Individuals with better oral health behaviors often demonstrate greater health awareness and healthier lifestyle habits, such as regular exercise and non‐smoking (Harada et al. 2005; Wiener et al. 2019). Conversely, poor periodontal status is frequently associated with unhealthy lifestyle habits, which may contribute to the development of MASLD. In addition to lifestyle factors, aging represents a shared risk factor for both periodontitis and MASLD. Age‐related changes and cellular senescence impair metabolic function and promote fatty metaplasia in the liver (Pinto et al. 2020), whereas reduced immune function with aging contributes to chronic inflammation and destruction of periodontal tissue (Ebersole et al. 2016). Moreover, metabolic diseases such as diabetes and obesity are well‐established risk factors for both periodontal disease and MASLD (Genco and Borgnakke (2013); Huang et al. 2020). Thus, the higher ORs of MASLD observed in individuals with poor periodontal health may largely reflect the overlap of shared risk factors, including adverse lifestyle behaviors, aging, and metabolic disorders.

However, periodontitis was associated with MASLD independently of common risk factors for these conditions in this study. Steatotic liver disease is considered a hepatic phenotype of metabolic syndrome, and the cardiometabolic criteria for MASLD are largely consistent with the diagnostic criteria for metabolic syndrome (Alberti et al. 2009). Periodontitis has been reported to be a risk factor for metabolic syndrome and its components, including obesity, hyperglycemia, hypertension, and dyslipidemia (Jepsen et al. 2020). The chronic inflammatory state induced by periodontitis is thought to promote insulin resistance and vascular endothelial inflammation. In addition, chronic inflammation may increase oxidative stress, and periodontitis has been shown to contribute to elevated oxidative stress (Guo et al. 2024). For these reasons, periodontitis is considered a risk factor for metabolic syndrome, diabetes, and obesity. By similar mechanisms, individuals with periodontitis may also be at higher risk for MASLD. Nevertheless, the causal nature and underlying mechanisms of the observed association in this study remain unclear, warranting further investigation.

In this study, the association between periodontitis and MASLD was observed only in women. One possible explanation is the influence of female sex hormones on this association. In women, hormonal secretion changes markedly across different life stages, altering disease risk accordingly (Bitzer 2009; Hogervorst et al. 2022). The participants in this study were aged 40–74 years, a range spanning the menopausal to geriatric period, during which hormonal shifts may affect both oral and systemic health. Estrogen, a key female hormone, plays multiple roles and is protective against the development of steatotic liver disease and other lifestyle‐related conditions (Eng et al. 2023). After menopause, the rapid decline in estrogen is associated with an increased risk of such diseases (De Paoli and Werstuck 2020). Estrogen is also involved in bone metabolism, and its deficiency promotes osteoclast differentiation, leading to reduced bone density and osteoporosis (Shi and Morgan 2024). As osteoporosis accelerates alveolar bone resorption, affected individuals are at greater risk of developing periodontitis and it progressing (Yu and Wang 2022). Moreover, estrogen has anti‐inflammatory properties (Knowlton and Lee 2012), and the loss of this effect after menopause may further predispose women to both MASLD and periodontitis. In this study, periodontitis was associated significantly with MASLD in women aged 50–59 years. This age range coincides with the menopausal transition, a period marked by a significant decline in female sex hormone secretion. This hormonal change may have influenced the association between periodontitis and MASLD, but the underlying mechanism remains unclear, as hormone secretion and its effects were not examined in this study.

Our interaction analysis revealed that the multiplicative interaction was significant, but the additive interaction was not. Furthermore, the multiplicative interaction was less than 1, implying that the association between periodontitis and MASLD was weaker in men. Sex differences in lifestyle habits also may have contributed to the observed sex difference in the association between periodontitis and MASLD. In general, women tend to have greater health awareness and adopt healthier lifestyle and oral health behaviors (Ministry of Health, Labour and Welfare, Labour and Welfare 2022, 2023). These social and cultural differences between men and women influence both oral and systemic health. The prevalence and severity of periodontal disease are consistently higher in men, regardless of age, race, or geographic region (Genco and Borgnakke 2013; Sangalli et al. 2023). Similarly, the prevalences of diabetes and obesity—established risk factors for periodontal disease—are also higher in men (Ministry of Health, Labour and Welfare 2023). Because lifestyle habits and oral health behaviors are closely linked to both steatotic liver disease and periodontal disease, men with poorer health behaviors are more likely to develop both conditions. In this study, the prevalence of MASLD was lower among women. This may have influenced the finding that the association between periodontitis and MASLD was stronger in women than in men. However, as the additive interaction was not significant, no marked absolute difference in risk was observed between men and women regarding the association between periodontitis and MASLD.

There are few reports on the association between periodontitis and MASLD, and the causal relationship and underlying mechanisms need to be clarified through longitudinal studies. As this study was conducted in a Japanese population, it is important to confirm that the findings are similar in other ethnic groups and regions. Future research should also account for socioeconomic and other potential confounding factors. Further investigation into sex differences in the association between periodontal disease and MASLD is particularly warranted.

## 5 Conclusion

In this study, periodontitis was associated with MASLD in women. Given the limited number of studies on this topic and the methodological limitations of this study, further research is needed to clarify the relationship between periodontitis and MASLD, including potential sex differences.

## Author Contributions

**Mizuki Saito:** conception, design, analysis, interpretation, and draft, critical revision of the manuscript. **Yoshihiro Shimazaki:** conception, design, interpretation, draft and critical revision of the manuscript. **Saori Yoshii:** conception, design, data acquisition, critical revision of the manuscript. **Tetuhito Kojima:** conception, design, data acquisition, critical revision of the manuscript. All authors gave their final approval and agree to be accountable for all aspects of the work.

## Ethics Statement

This study was approved by the Institutional Review Board of Aichi Gakuin University School of Dentistry (approval number 618), and it was carried out in accordance with the Helsinki Declaration.

## Conflicts of Interest

The authors declare no conflicts of interest.

## Supporting information


Supporting File:

## Data Availability

The data that support the findings of this study are available from the Aichi Health Promotion Foundation, but restrictions apply to the availability of these data, which were used under license for the current study, and so are not publicly available. Data are, however, available from the authors upon reasonable request and with the permission of the Aichi Health Promotion Foundation.

## References

[cre270355-bib-0001] Alberti, K. G. , R. H. Eckel , S. M. Grundy , et al. 2009. “Harmonizing the Metabolic Syndrome: A Joint Interim Statement of the International Diabetes Federation Task Force on Epidemiology and Prevention; National Heart, Lung, and Blood Institute; American Heart Association; World Heart Federation; International Atherosclerosis Society; and International Association for the Study of Obesity.” Circulation 120, no. 16: 1640–1645. 10.1161/circulationaha.109.192644. 19805654

[cre270355-bib-0002] Bitzer, J. 2009. “Progesterone, Progestogens and Psychosomatic Health of the Climacteric Woman.” Maturitas 62, no. 4: 330–333. 10.1016/j.maturitas.2008.10.008. 19091497

[cre270355-bib-0003] Ebersole, J. L. , C. L. Graves , O. A. Gonzalez , et al. 2016. “Aging, Inflammation, Immunity and Periodontal Disease.” Periodontology 2000 72, no. 1: 54–75. 10.1111/prd.12135. 27501491

[cre270355-bib-0004] Eguchi, Y. , H. Hyogo , M. Ono , et al. 2012. “Prevalence and Associated Metabolic Factors of Nonalcoholic Fatty Liver Disease in the General Population From 2009 to 2010 in Japan: A Multicenter Large Retrospective Study.” Journal of Gastroenterology 47, no. 5: 586–595. 10.1007/s00535-012-0533-z. 22328022

[cre270355-bib-0005] Eng, P. C. , R. Forlano , T. Tan , P. Manousou , W. S. Dhillo , and C. Izzi‐Engbeaya . 2023. “Non‐Alcoholic Fatty Liver Disease in Women—Current Knowledge and Emerging Concepts.” JHEP Reports 5, no. 10: 100835. 10.1016/j.jhepr.2023.100835. 37771547 PMC10522907

[cre270355-bib-0006] Genco, R. J. , and W. S. Borgnakke . 2013. “Risk Factors for Periodontal Disease.” Periodontology 2000 62, no. 1: 59–94. 10.1111/j.1600-0757.2012.00457.x. 23574464

[cre270355-bib-0007] Guo, S. , L. Fu , C. Yin , et al. 2024. “ROS‐Induced Gingival Fibroblast Senescence: Implications in Exacerbating Inflammatory Responses in Periodontal Disease.” Inflammation 47, no. 6: 1918–1935. 10.1007/s10753-024-02014-5. 38630168

[cre270355-bib-0008] Harada, S. , R. Akhter , K. Kurita , et al. 2005. “Relationships Between Lifestyle and Dental Health Behaviors in a Rural Population in Japan.” Community Dentistry and Oral Epidemiology 33, no. 1: 17–24. 10.1111/j.1600-0528.2004.00189.x. 15642043

[cre270355-bib-0009] Heidari, S. , T. F. Babor , P. De Castro , S. Tort , and M. Curno . 2016. “Sex and Gender Equity in Research: Rationale for the SAGER Guidelines and Recommended Use.” Research Integrity and Peer Review 1: 2. 10.1186/s41073-016-0007-6. 29451543 PMC5793986

[cre270355-bib-0010] Hogervorst, E. , J. Craig , and E. O'donnell . 2022. “Cognition and Mental Health in Menopause: A Review.” Best Practice & Research Clinical Obstetrics & Gynaecology 81: 69–84. 10.1016/j.bpobgyn.2021.10.009. 34969617

[cre270355-bib-0011] Huang, T. , J. Behary , and A. Zekry . 2020. “Non‐Alcoholic Fatty Liver Disease: A Review of Epidemiology, Risk Factors, Diagnosis and Management.” Internal Medicine Journal 50, no. 9: 1038–1047. 10.1111/imj.14709. 31760676

[cre270355-bib-0012] Jepsen, S. , J. Suvan , and J. Deschner . 2020. “The Association of Periodontal Diseases With Metabolic Syndrome and Obesity.” Periodontology 2000 83, no. 1: 125–153. 10.1111/prd.12326. 32385882

[cre270355-bib-0013] Knowlton, A. A. , and A. R. Lee . 2012. “Estrogen and the Cardiovascular System.” Pharmacology & Therapeutics 135, no. 1: 54–70. 10.1016/j.pharmthera.2012.03.007. 22484805 PMC5688223

[cre270355-bib-0014] Kolay, E. , A. Bykowska‐Derda , S. Abdulsamad , et al. 2021. “Self‐Reported Eating Speed Is Associated With Indicators of Obesity in Adults: A Systematic Review and Meta‐Analysis.” Healthcare 9, no. 11: 1559. 10.3390/healthcare9111559. 34828605 PMC8619990

[cre270355-bib-0015] Kuraji, R. , S. Sekino , Y. Kapila , and Y. Numabe . 2021. “Periodontal Disease‐Related Nonalcoholic Fatty Liver Disease and Nonalcoholic Steatohepatitis: An Emerging Concept of Oral‐Liver Axis.” Periodontology 2000 87, no. 1: 204–240. 10.1111/prd.12387. 34463983 PMC8456799

[cre270355-bib-0016] Kuroe, K. , M. Furuta , K. Takeuchi , et al. 2021. “Association Between Periodontitis and Fibrotic Progression of Non‐Alcoholic Fatty Liver Among Japanese Adults.” Journal of Clinical Periodontology 48, no. 3: 368–377. 10.1111/jcpe.13415. 33368494

[cre270355-bib-0017] Maurice, J. , and P. Manousou . 2018. “Non‐Alcoholic Fatty Liver Disease.” Clinical Medicine 18, no. 3: 245–250. 10.7861/clinmedicine.18-3-245. 29858436 PMC6334080

[cre270355-bib-0018] Ministry of Health, Labour and Welfare . 2022. “Survey of Dental Diseases”. https://www.mhlw.go.jp/content/10804000/001112405.pdf.

[cre270355-bib-0019] Ministry of Health, Labour and Welfare . 2023. “National Health and Nutrition Survey”. https://www.mhlw.go.jp/content/001435384.pdf.

[cre270355-bib-0020] De Paoli, M. , and G. H. Werstuck . 2020. “Role of Estrogen in Type 1 and Type 2 Diabetes Mellitus: A Review of Clinical and Preclinical Data.” Canadian Journal of Diabetes 44, no. 5: 448–452. 10.1016/j.jcjd.2020.01.003. 32127295

[cre270355-bib-0021] Pham, T. A. , M. Ueno , K. Shinada , T. Yanagisawa , F. A. Wright , and Y. Kawaguchi . 2011. “Periodontal Disease and Related Factors Among Vietnamese Dental Patients.” Oral Health & Preventive Dentistry 9, no. 2: 185–194. 21842021

[cre270355-bib-0022] Pinto, C. , E. Ninfole , L. Gaggiano , A. Benedetti , M. Marzioni , and L. Maroni . 2020. “Aging and the Biological Response to Liver Injury.” Seminars in Liver Disease 40, no. 3: 225–232. 10.1055/s-0039-3402033. 31887774

[cre270355-bib-0023] Rinella, M. E. , J. V. Lazarus , V. Ratziu , et al. 2023. “A Multisociety Delphi Consensus Statement on New Fatty Liver Disease Nomenclature.” Hepatology 78, no. 6: 1966–1986. 10.1097/hep.0000000000000520. 37363821 PMC10653297

[cre270355-bib-0024] Sangalli, L. , L. C. Souza , A. Letra , L. Shaddox , and E. Ioannidou . 2023. “Sex as a Biological Variable in Oral Diseases: Evidence and Future Prospects.” Journal of Dental Research 102, no. 13: 1395–1416. 10.1177/00220345231197143. 37967405

[cre270355-bib-0025] Sato, S. , Y. Kamata , T. Kessoku , et al. 2022. “A Cross‐Sectional Study Assessing the Relationship Between Non‐Alcoholic Fatty Liver Disease and Periodontal Disease.” Scientific Reports 12, no. 1: 13621. 10.1038/s41598-022-17917-2. 35948584 PMC9365789

[cre270355-bib-0026] Shi, V. , and E. F. Morgan . 2024. “Estrogen and Estrogen Receptors Mediate the Mechanobiology of Bone Disease and Repair.” Bone 188: 117220. 10.1016/j.bone.2024.117220. 39106937 PMC11392539

[cre270355-bib-0027] Shin, H. S. , M. H. Hong , J. Y. Moon , and S. J. Sim . 2022. “Periodontal Disease Could Be a Potential Risk Factor for Non‐Alcoholic Fatty Liver Disease: An 11‐Year Retrospective Follow‐up Study.” Clinical Oral Investigations 26, no. 8: 5503–5514. 10.1007/s00784-022-04518-6. 35556175

[cre270355-bib-0028] Shine, B. K. , M. Son , S. Y. Moon , and S. H. Han . 2024. “Metabolic Dysfunction‐Associated Steatotic Liver Disease and the Risk of Chronic Periodontitis: A Nationwide Cohort Study.” Nutrients 17, no. 1: 125. 10.3390/nu17010125. 39796559 PMC11723414

[cre270355-bib-0029] Suzuki, K. , N. Tamaki , M. Kurosaki , et al. 2024. “Concordance Between Metabolic Dysfunction‐Associated Steatotic Liver Disease and Nonalcoholic Fatty Liver Disease.” Hepatology Research 54, no. 6: 600–605. 10.1111/hepr.14011. 38234088

[cre270355-bib-0030] Tamber, S. S. , P. Bansal , S. Sharma , R. B. Singh , and R. Sharma . 2023. “Biomarkers of Liver Diseases.” Molecular Biology Reports 50, no. 9: 7815–7823. 10.1007/s11033-023-08666-0. 37482588

[cre270355-bib-0031] Tan, L. , Y. He , T. Wang , X. Gao , W. Fan , and B. Fan . 2024. “A Mendelian Randomization Study Between Chronic Periodontitis and Non‐Alcoholic Fatty Liver Disease.” Journal of Periodontal Research 59, no. 2: 346–354. 10.1111/jre.13218. 38102730

[cre270355-bib-0032] Weintraub, J. A. , G. Lopez Mitnik , and B. A. Dye . 2019. “Oral Diseases Associated With Nonalcoholic Fatty Liver Disease in the United States.” Journal of Dental Research 98, no. 11: 1219–1226. 10.1177/0022034519866442. 31369716 PMC6755718

[cre270355-bib-0033] Wiener, R. C. , R. Bhandari , A. K. Trickett Shockey , and C. Waters . 2019. “Dental Care Utilization Among Veterans by Smoking Status.” International Journal of Dentistry 2019: 3419805. 10.1155/2019/3419805. 30881454 PMC6383398

[cre270355-bib-0034] Williams, J. , L. Allen , K. Wickramasinghe , B. Mikkelsen , N. Roberts , and N. Townsend . 2018. “A Systematic Review of Associations Between Non‐Communicable Diseases and Socioeconomic Status Within Low‐ and Lower‐Middle‐Income Countries.” Journal of Global Health 8, no. 2: 020409. 10.7189/jogh.08.020409. 30140435 PMC6076564

[cre270355-bib-0035] World Health Organization . 1997. “Oral Health Surveys, Basic Methods. Fourth ed." Geneva. Wold Health Organization.

[cre270355-bib-0036] World Health Organization International Agency for Research on Cancer . 2022. “Global Cancer Observatory” https://gco.iarc.fr/en.

[cre270355-bib-0037] Yan, C. , J. Bao , and J. Jin . 2024. “Exploring the Interplay of Gut Microbiota, Inflammation, and LDL‐Cholesterol: A Multiomics Mendelian Randomization Analysis of Their Causal Relationship in Acute Pancreatitis and Non‐Alcoholic Fatty Liver Disease.” Journal of Translational Medicine 22, no. 1: 179. 10.1186/s12967-024-04996-0. 38374155 PMC10875775

[cre270355-bib-0038] Yoneda, M. , S. Naka , K. Nakano , et al. 2012. “Involvement of a Periodontal Pathogen, Porphyromonas Gingivalis on the Pathogenesis of Non‐Alcoholic Fatty Liver Disease.” BMC Gastroenterology 12, no. 16: 16. 10.1186/1471-230x-12-16. 22340817 PMC3305584

[cre270355-bib-0039] Younossi, Z. M. 2019. “Non‐Alcoholic Fatty Liver Disease—A Global Public Health Perspective.” Journal of Hepatology 70, no. 3: 531–544. 10.1016/j.jhep.2018.10.033. 30414863

[cre270355-bib-0040] Younossi, Z. M. , M. Kalligeros , and L. Henry . 2025. “Epidemiology of Metabolic Dysfunction‐Associated Steatotic Liver Disease.” Supplement, Clinical and Molecular Hepatology 31, no. Suppl: S32–S50. 10.3350/cmh.2024.0431. 39159948 PMC11925440

[cre270355-bib-0041] Younossi, Z. M. , J. M. Paik , M. Stepanova , J. Ong , S. Alqahtani , and L. Henry . 2024. “Clinical Profiles and Mortality Rates Are Similar for Metabolic Dysfunction‐Associated Steatotic Liver Disease and Non‐Alcoholic Fatty Liver Disease.” Journal of Hepatology 80, no. 5: 694–701. 10.1016/j.jhep.2024.01.014. 38286339

[cre270355-bib-0042] Younossi, Z. M. , S. Zelber‐Sagi , L. Henry , and L. H. Gerber . 2023. “Lifestyle Interventions in Nonalcoholic Fatty Liver Disease.” Nature Reviews Gastroenterology & Hepatology 20, no. 11: 708–722. 10.1038/s41575-023-00800-4. 37402873

[cre270355-bib-0043] Yu, B. , and C. Y. Wang . 2022. “Osteoporosis and Periodontal Diseases—An Update on Their Association and Mechanistic Links.” Periodontology 2000 89, no. 1: 99–113. 10.1111/prd.12422. 35244945 PMC9067601

[cre270355-bib-0044] Zhang, Z. , Q. Zheng , Y. Liu , G. Chen , and Y. Li . 2025. “Association Between Periodontitis and Mortality in Participants With Metabolic Dysfunction‐Associated Steatotic Liver Disease: Results From NHANES.” BMC Oral Health 25, no. 1: 567. 10.1186/s12903-025-05959-7. 40223086 PMC11995466

